# Progress in reeducating tumor-associated macrophages in tumor microenvironment

**DOI:** 10.1007/s12672-024-01186-8

**Published:** 2024-07-26

**Authors:** Yiming Zhao, Qianyang Ni, Weijian Zhang, Suyang Yu

**Affiliations:** Department of Gastrointestinal Surgery, The Third Hospital of Hebei Medical University, No.139, Ziqiang Road, Qiaoxi District, Shijiazhuang, 050051 China

**Keywords:** Tumor-associated macrophages, Tumour microenvironment, Macrophage polarization, Nanoparticles, Photodynamic therapy

## Abstract

Malignant tumor, one of the most threatening diseases to human health, has been comprehensively treated with surgery, radiotherapy, chemotherapy and targeted therapy, but the prognosis has not always been ideal. In the past decade, immunotherapy has shown increased efficacy in tumor treatment; however, for immunotherapy to achieve its fullest potential, obstacles are to be conquered, among which tumor microenvironment (TME) has been widely investigated. In remodeling the tumor immune microenvironment to inhibit tumor progression, macrophages, as the most abundant innate immune population, play an irreplaceable role in the immune response. Therefore, how to remodel TME and alter the recruitment and polarization status of tumor-associated macrophages (TAM) has been of wide interest. In this context, nanoparticles, photodynamic therapy and other therapeutic approaches capable of affecting macrophage polarization have emerged. In this paper, we categorize and organize the existing means and methods for reprogramming TAM to provide ideas for clinical application of novel tumor-related therapies.

## Introduction

Malignant tumours are currently the biggest killer of human health worldwide [1], and the current treatment is a combination of surgery and radiotherapy [2]. Immunotherapy has emerged as an effective anti-tumour strategy [3], working by activating the patient's own immune system to self-recognize and destroy tumours while avoiding serious damage to normal tissues and cells [4, 5]. However, only a small proportion of patients respond to immunotherapy due to immunosuppression caused by the tumour microenvironment (TME). Therefore, how to remodel the tumour microenvironment to inhibit tumour angiogenesis and metastasis is of great importance for tumour treatment [6–8]. The TME is composed of tissue-resident cells and recruited immune cells, which constitute up to 50% of the tumour mass in some solid tumours such as breast cancer [9], and macrophages are usually the most abundant of these cells. There is increasing evidence that some macrophage subpopulations can effectively influence tumour progression and interfere with the treatment of almost all types of cancer [10]. More specifically, M2-type TAMs in tumour-associated macrophages play a role in TME by promoting immunosuppression and reducing drug efficacy [11]. Therefore, more and more researchers are focusing on the biological properties of macrophages with the ultimate goal of overcoming the limitations of current therapeutic approaches. Several aspects of current interest include: (I) The complexity and diversity of macrophages and how to identify different macrophage types. (II) How different types of macrophages promote or inhibit tumour progression. (III) The possibility of rational manipulation of macrophages to achieve anti-tumour responses while preserving normal tissue. (IV) The impact of the above manipulations on patient prognosis [12].

## Phenotype and function of TAM

**Fig. 1:**
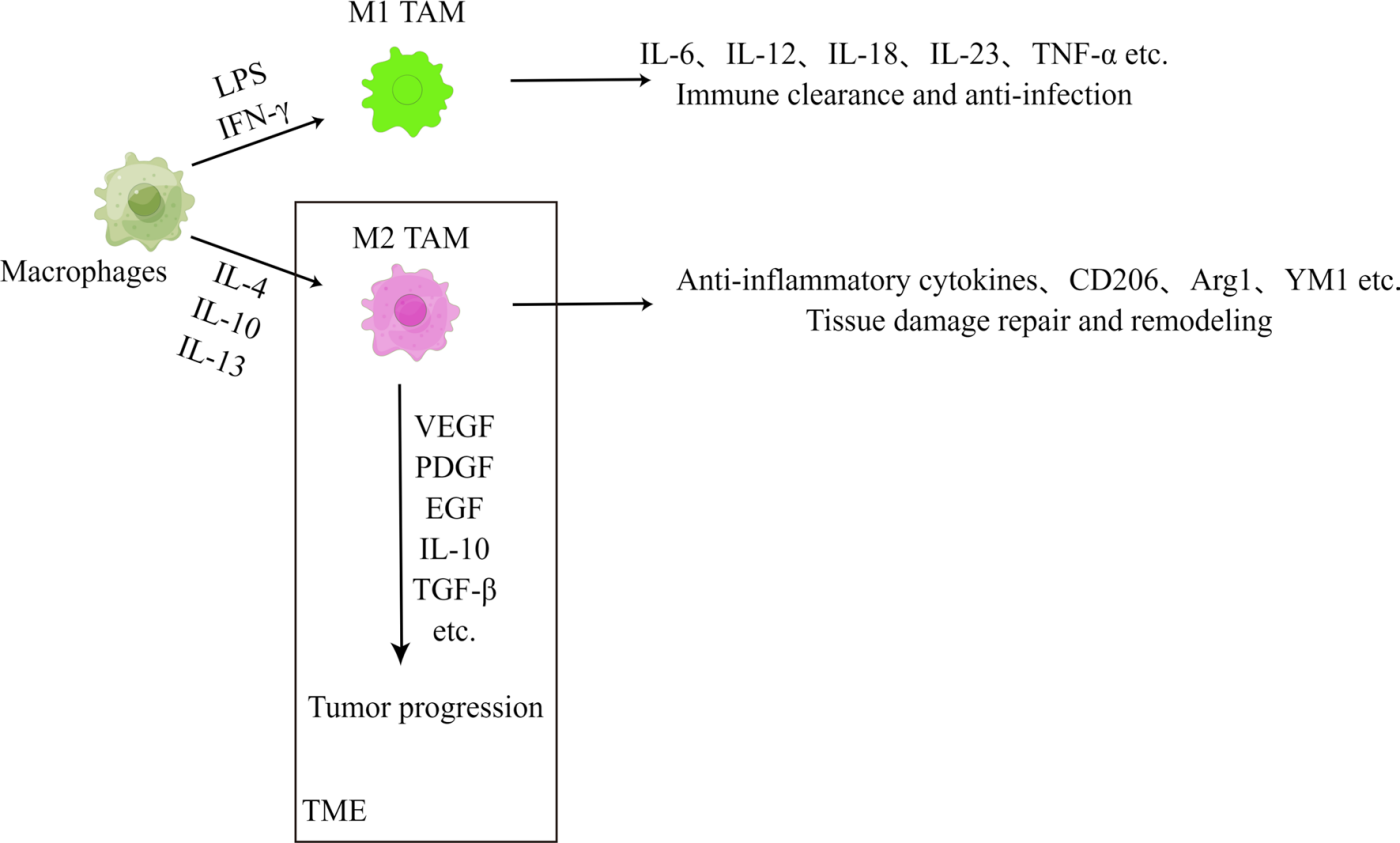
TAM’s phenotype and function. (By Figdraw) LPS: lipopolysaccharide; IFN-γ: interferon γ; CD206: the mannose receptor; Arg1: arginase-1;YM1: chitinase 3

It has been shown that high infiltration of TAM in human tumors is associated with poor clinical outcomes [13]. In contrast, TAM are highly heterogeneous in phenotype and function [13], and specific subpopulations of TAM have distinct roles in cancer progression and antitumour immunity. TAMs are highly plastic and undergo polarisation activation in response to different signalling stimuli. Activated macrophages can be divided into two subpopulations, the classically activated M1-type TAM and the alternatively activated M2-type TAM [14–17]. Macrophages activated by lipopolysaccharide (LPS) and interferon γ (IFN-γ) are termed classically activated macrophages, also known as M1-type TAM, which mainly secrete pro-inflammatory factors such as IL-12, IL-6, IL-18, IL-23 and TNF-α (Fig. 1). Therefore, M1-type TAM can perform immune clearance functions against pathogens and tumours and play an important role in the body’s defence against infection. Macrophages activated by IL-4, IL-10 and IL-13 are called alternatively activated macrophages, also known as M2-type TAMs. IL-4-induced bone marrow-derived haematopoietic stem cells are M2-type TAMs that secrete anti-inflammatory cytokines such as IL-10 and IL-1 receptor antagonists, and overexpress the mannose receptor (CD206), arginase-1 (Arg1) and chitinase 3 (YM1) (Fig. 1), which play important roles in repairing tissue damage and remodelling in late inflammation. During tumour progression, TAMs may be more inclined to the M2 polarized state [18] and can secrete various growth factors such as VEGF, PDGF, EGF, chemokines and cytokines such as IL-10, TGF-β, CCL2 and CXCL12 [19]; thus, the tissue trophic function of M2-type TAMs instead promotes tumour progression [20].

It has been shown that M2-type macrophages can be further subdivided into four subpopulations, termed M2a, M2b, M2c and M2d, based on the stimuli they receive and the phenotypes and functions they produce [21]. IL-4 and IL-13 induce macrophage transformation to M2a, which secretes VEGF and CCL18, which together induce angiogenesis and promote tumour cell migration and invasion in breast cancer [22]. LPS and IC are considered classical inducers of M2b [23], and only a small number of M2b macrophages are detected in the early stages. However, as the tumour progresses, the number of M2b cells increases and gradually replaces the M1 cell population [24] and promotes tumour progression by secreting factors such as IL-10, IL-6 and IL-1 [21]. M2c are macrophages stimulated by IL-10, TGF-β or glucocorticoids. Immunomodulation is a hallmark of M2c macrophages, which have the ability to depolarise macrophages into M1 and produce pro-inflammatory cytokines. They can also support tumour growth by inducing angiogenesis [21]. IL-6 and LIF have been shown to polarise macrophages into a new M2 subtype called M2d. M2d macrophages promote tumour progression through two main mechanisms: First, M2d cells are capable of producing pro-tumourigenic factors such as IL-10 and TGF-β. In addition, VEGF and MMP9 produced by M2d are expected to induce angiogenesis and extracellular matrix degradation, thereby promoting tumour metastasis [21].

Single-cell RNA sequencing (scRNA-seq) can greatly improve the understanding of cellular function and heterogeneity by providing more comprehensive transcriptomic information at single-cell resolution. ScRNA-seq technology has emerged as a valuable tool for dissecting the cellular transcriptome, helping to uncover cell types and their functional status, which is critical for determining patient prognosis. Some studies have summarised the key features of TAM subpopulations in human cancers, but at the same time it has been found that due to the severe heterogeneity of TAM populations in different tumour types. For example, colorectal cancer contains multiple subtypes of TAMs such as TREM2 + , CIQC + , SPP1 + , CTSB + , APOC1 + and so on at the same time, which is a great challenge for how to effectively identify the M2 type of TAMs and re-educate them [25, 26].

The presence of different TAMs in different tumours makes it even more important for researchers to select the right drug and target when choosing the appropriate therapy, which helps to develop appropriate treatments for specific tumour microenvironments.

## TAM plasticity and tumors

Under certain circumstances, the two types of TAM can be interconverted: Stimulation of M2-type TAM with M1-type inducers can induce repolarisation to M1 and vice versa, suggesting that macrophages are plastic. The JAK/STAT pathway has been shown to promote TAM towards M2-like polarisation, which inhibits the recruitment of NK and CD8 + T cells and ultimately promotes the proliferation of renal clear cell carcinoma (KIRC) [27]. Cytokines such as LPS or IFN-γ contribute to M1-type TAM activation by activating the ICAM1-PI3K-Akt-Notch1 and JAK1-STAT1-Caspase pathways to defend against pathogens, which promote inflammatory responses and tumour cell apoptosis [28]. In contrast, IL-4 and IL-13 activate the STAT6 pathway through IL-4Rα [28–30], which drives macrophage activation towards the M2 type. In addition, other cytokines such as IL-10 can also regulate polarisation towards M2-type TAM through activation of STAT3 by IL-10 receptors, further accelerating TME remodelling and promoting tumour development and metastasis. Recently, Huang et al. identified spatial heterogeneity of TAMs in tumours, with D68 + IRF8 + TAMs (M1) wrapped around the inner regions of tumour masses and CD68 + CD163 + CD206 + TAMs (M2) enriched in the peritumoural regions. This distribution pattern suggests that M2 TAMs exert immunosuppressive effects and support tumour invasion, whereas M1 TAMs may be involved in necrosis of the tumour core [31]. Therefore, targeting M2 TAMs and depleting them in the TME, or reversing M2 TAMs to M1 phenotype, directly enhancing their cytotoxicity and indirectly stimulating cytotoxic T cells to eliminate tumour cells, are potential strategies for anti-tumour immunotherapy.

It is also believed that in the early stages of tumour development, TAMs are induced into the M1 phenotype by the action of pro-inflammatory cytokines. Once the tumour is established, TAMs are "re-educated" into the M2 phenotype, which supports tumour growth and promotes tumour progression through the production of tumour growth factors (e.g. EGF, FGF, TGFb, PDGF) and pro-angiogenic molecules (e.g. TGFb, PDGF). Accordingly, we believe that the goal of re-educating TAMs can be added to the goal of stopping the process of conversion of M1 to M2 type TAMs after tumour formation.

The most important markers of TAMs have been identified by studying tumours at different sites: CD163, CD204 (MSR1), CD206 (MRC1), MARCO, SIGLEC1 (CD169), stabilising protein-1 (Stab1) and Tie2 (TEK). These are M2 markers associated with poor prognosis in cancer patients. According to available evidence, CD206 and CD163 are the most common markers of M2-type TAMs, which are associated with metastasis and poor prognosis in many cancers [26].

## Treatment strategies targeting tumor-associated macrophages

Macrophages are an important component of the human immune system and can migrate to tumour sites by natural chemotaxis [33] and release a variety of cytokines to regulate the TME [19]. They also possess the antigen-presenting ability to recognise and engulf cancer cells, thereby initiating an immune response [34]. Therefore, macrophages have become an important tool for tumour therapy and macrophage-based tumour treatment strategies have been widely investigated. Current potential strategies to inhibit macrophage function in the TME include: (I) Blocking TAM accumulation at tumour sites by eliminating the presence of TAM or inhibiting their recruitment. The most established approach to reduce TAM survival is to block the CSF1/CSF1R axis. In addition, disruption of CCL2/CCR2 signalling also reduces the recruitment of inflammatory monocytes from the bone marrow and peripheral blood to the tumour site and significantly reduces macrophage infiltration [31–33]. (II) Re-taming TAMs to an anti-tumour phenotype. The high plasticity of TAMs is exploited to convert them from M2 to M1 phenotype. A number of drugs and therapeutic tools are currently available for such approaches.

### TAM-targeted nanoparticles for antitumor therapy

**Fig. 2:**
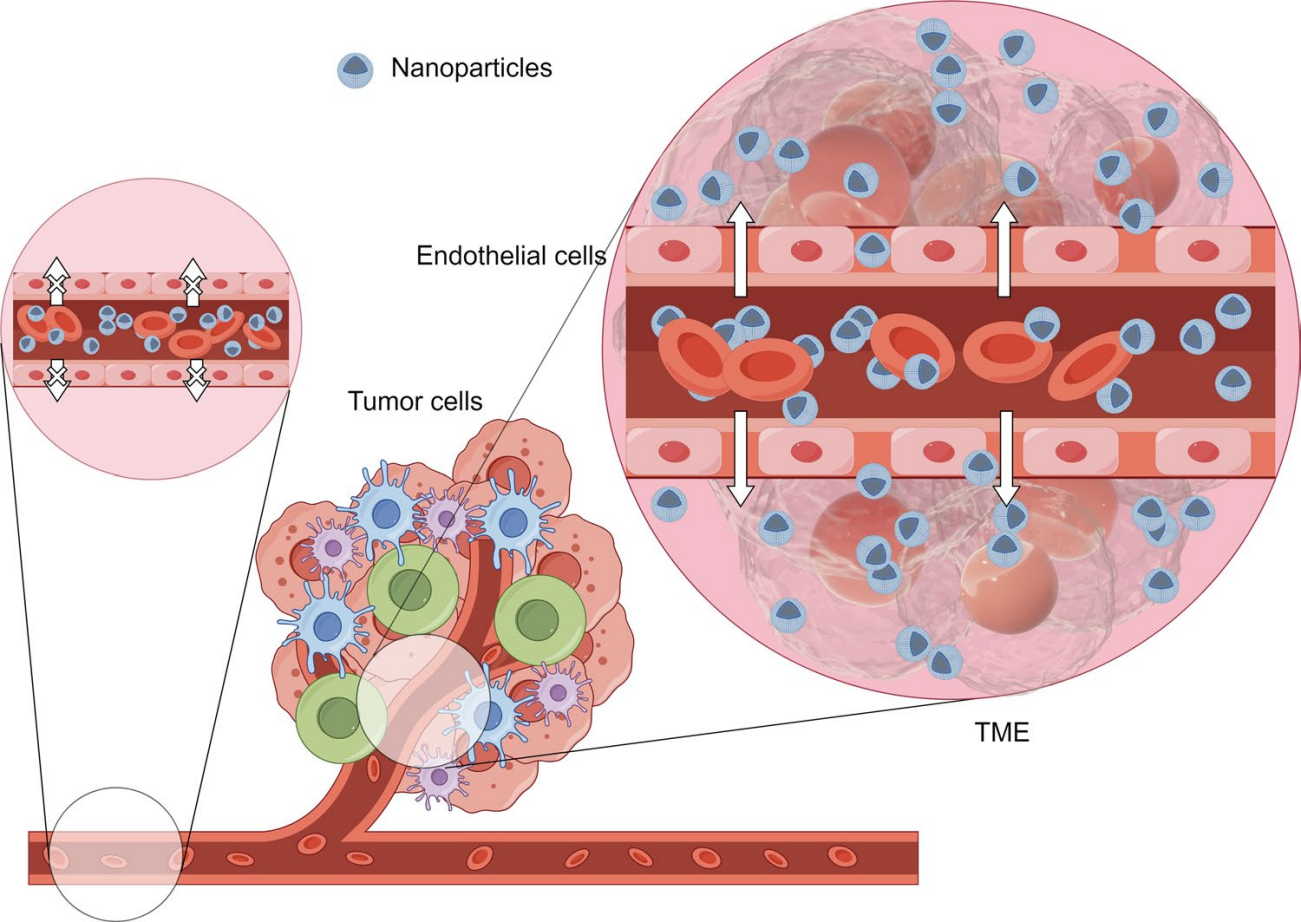
The EPR effect in TME. (By Figdraw)

The International Union of Pure and Applied Chemistry (IUPAC) defines a nanoparticle as any particle of submicron size [35]. Such nanoparticles have several characteristics: (I) Relatively high surface area. (II) Targeting by modification to reduce systemic toxicity. (III) Relatively stable structure that prevents early drug degradation. Targeting of nanoparticles to tumour cells can be divided into two types: Passive targeting and active targeting. Traditionally, it is believed that nanoparticles are easily retained in tumour sites due to the significantly higher growth rate of tumours than normal tissue, poor arrangement between tumour vascular endothelial cells and slow blood flow within the tumour, a phenomenon known as the Enhanced Permeability and Retention (EPR) effect [36] (Fig. 2). The EPR effect is the theoretical basis for passive targeting of nanoparticles. However, in recent years it has been suggested that transcytosis is the dominant mechanism for passive targeting of nanoparticles [37]. Surface-targeted modification of nanoparticles can convert them into active targeting capability.

**Fig. 3:**
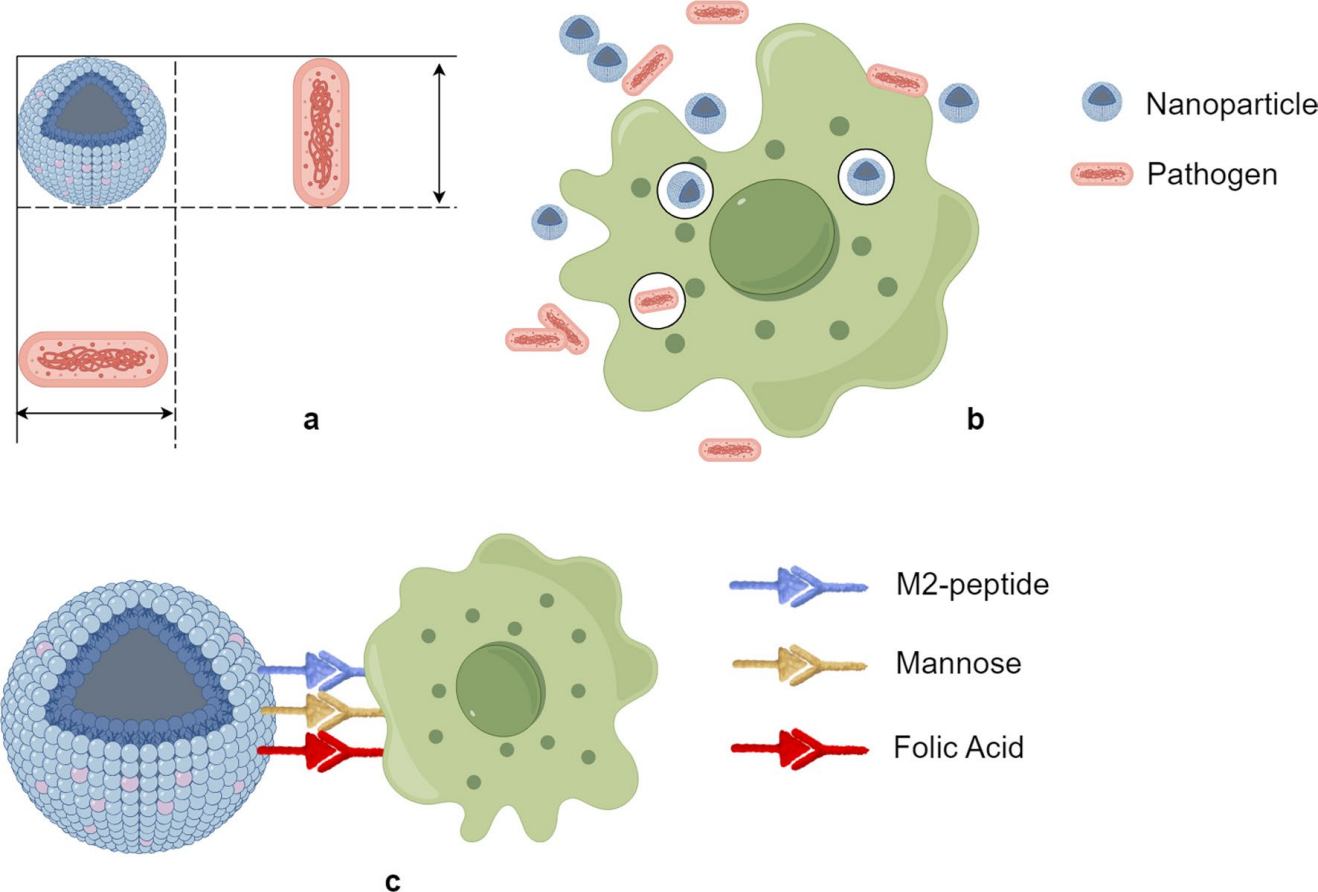
The schematic diagram of active/passive targeting nanoparticles. (By Figdraw) **A** Tailoring the nanoparticles to be close to the size of the pathogen. **B** Nanoparticles similar in size to pathogens can be captured along with them by macrophages. **C** Active targeting can be achieved by enabling nanoparticles to carry specific ligands such as M2-peptide, mannose and folate, which could bind to TAM-specific receptors

Macrophages can also successfully load chemotherapeutic agents or drug-loaded nanoparticles through their phagocytosis for targeted delivery to tumour sites for effective anti-tumour therapy [38, 39]. It is known that large particles are cleared by the mononuclear phagocyte system and the liver, so nanoparticles can be tailored to larger sizes and shapes similar to pathogens, making macrophages more inclined to take them up. Active targeting nanoparticles also bind to TAM-specific receptors by modifying some ligands, common ones being M2 peptide, mannose and folic acid [40] (Fig. 3). Alternatively, nanoparticles carrying drugs or proteins, such as targeted kinase inhibitors or CSF-1R inhibitors, can also induce TAM polarisation towards the M1 type [41], thereby inhibiting tumour cells. In addition to entrapped anti-tumour drugs, some of the nanoparticle's own materials are also known to have the effect of reprogramming TAM to inhibit tumour proliferation through a different pathway.

According to the different substrates, nanoplatforms can generally be classified into polymeric nanoparticles, lipid-based nanocarriers, inorganic nanomaterials, etc. Macrophages have multiple functions as drug carriers: On the one hand, they have a long blood half-life in vivo; on the other hand, integrins on the surface of macrophages can bind to tumour vascular cell adhesion molecule 1 (VCAM-1) and thus have the ability to specifically target tumours [42].

#### Lipid based nanomaterials

**Fig. 4:**
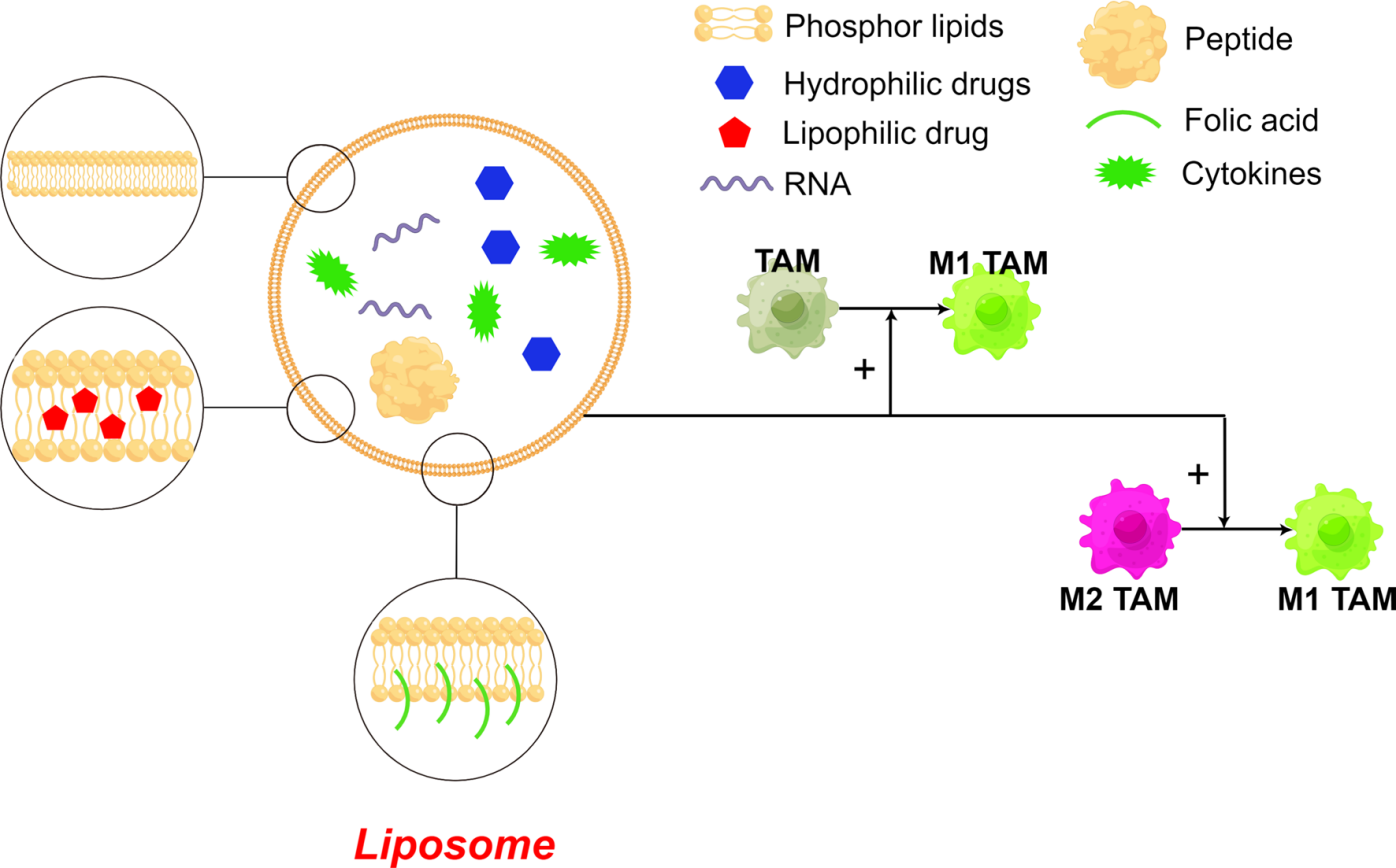
Functions and characteristics of liposomes. (By Figdraw) Bionic structure: core encapsulated hydrophilic drug, lipid layer encapsulated hydrophobic drug; Size: 40–50 nm, same as natural exosomes

Liposomes, a closed bilayer phospholipid membrane structure, have been widely investigated as therapeutic carriers due to their biocompatibility, biodegradability, low toxicity, ease of surface modification and higher cargo encapsulation capacity [28]. They have particular advantages. (I) They have a bionanostructure in which the liposome core and lipid layer can encapsulate hydrophilic drugs, which can overcome the problem of inconvenient loading of drugs with different solubilities. (II) With the size of 40–150 nm, the same as that of natural exosomes, they are widely used as biological nanocarriers in oncological therapy [43, 44] (Fig. 4).

Given the properties of nanoliposomes, they can be used to carry different drugs such as bisphosphonates, different RNAs, cytokines, etc. to polarise TAMs. Sousa, S. found that liposomes encapsulating zoledronic acid (L-ZOL) altered the ratio of TAM in breast cancer, increasing M1-type TAM and thus promoting apoptosis in cancer cells [45]. The nanoliposomes constructed by Ramesh, A. et al. carrying the CSF1R inhibitor BLZ945 and the CD47-SIRPα inhibitor SHP099 significantly downregulated CD206 expression in the macrophage cell line raw264.7, increased the M1/M2 ratio and inhibited tumour proliferation [46]. And then Ramesh, A. et al. invented a lipid-based supramolecular nanoparticle that can carry two kinase inhibitors with different structural properties to promote repolarisation of M2-type TAM through sustained inhibition of CSF1R and MAPK signalling pathways. This nanostructure allows better accumulation of inhibitors in tumours and precise delivery to the same type of TAM [47], which in turn inhibits tumour cell proliferation. Hypoxia-inducible factor-1α (HIF-1α) is a detrimental tumour-promoting factor in TME. Nour Shobaki. et al. developed a lipid nanocarrier containing siRNA capable of blocking HIF-1α and successfully promoted the secretion of TNF-α and IFN-γ in TME, and a significant increase in the number of CD169 + TAMs showed its anti-tumour effect [48]. Resiquimod (R848) is an immune system activator that has been shown to stimulate M1-type TAM and promote repolarisation to M2-type. One research group found that liposomes encapsulating R848 in combination with anti-EGFR antibodies were able to repolarise TAM to the M1 type and enhance the anti-tumour effects of therapeutic antibodies, thereby significantly inhibiting tumour growth [49].

Cytokine and chemokine therapy also benefits from nanocarriers. CCL2 and CCL5 are two major chemokines that play an important role in TAM infiltration and induction of M2-type differentiation, and an antibody that efficiently and specifically binds and inhibits CCL2 and CCL5 (BisCCL2/5i) was developed. The mRNA encoding BisCCL2/5i was encapsulated in lipid nanoparticles, and the BisCCL2/5i mRNA nanoplatform significantly induced TAM differentiation to the M1 type and reduced immunosuppression in the TME [50]. Tang introduced CD47-derived self-peptide and galactose ligands onto liposomes, and covered the surface with cleavable phospholipid-polyethylene glycol, which protected galactose from hepatic clearance and promoted liposome accumulation in tumours. Loaded adriamycin selectively eliminates M2 TAM to improve the efficacy of anti-tumour therapy [51]. Notably, Doxil® was the first nanomedicine approved by the FDA and liposomal cisplatin was approved by the European Medicines Evaluation Agency (EMEA) for the treatment of pancreatic cancer [52].

Lipid nanoemulsions can be prepared using ultrasound and other devices. Multi-lipid nanoemulsions tend to have low dispersion, and lipid nanoemulsions can contain hydrophobic drug molecules within oil droplets. Ye et al. constructed a lipid nanoemulsion carrying isoflavones, called neoflavonoids, which were able to convert M2 TAM to M1 type in vivo, thereby inhibiting cancer cell proliferation [53]. Li prepared lipid nanoemulsions (LNs) for the co-delivery of paclitaxel (PTX), docosahexaenoic acid (DHA) and folic acid (FA) decorators (PTXA/DHA-FA-LNs), which are more readily taken up by M2 TAM in vitro and can use their phagocytosis to concentrate the drug in cancer nests and improve antitumour efficacy [54].

#### Polymeric nanoparticles

**Fig. 5:**
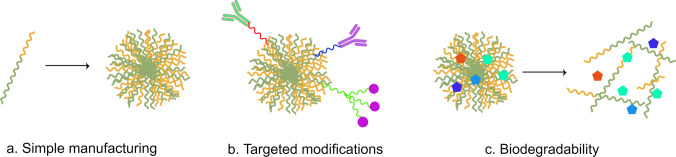
Functions and characteristics of polymers. (By Figdraw) **A** Relatively simple manufacturing techniques offer the possibility of making off-the-shelf nanomedicines. **B** Facilitates targeted modifications. **C** Relatively biodegradable and biocompatible, especially with natural polymer nanoparticles, which are also biologically safe

Polymeric nanoparticles are one of the most important nanoscale formulations, and many synthetic or natural polymers can be used as NP constructs for the delivery of anticancer molecules, and most of them are biodegradable and can be easily attached to hyaluronic acid and other targeting ligands for targeted drug delivery to different types of cancers [55]. Polymeric materials have several outstanding advantages: (I) Relatively simple fabrication techniques. (II) Ease of targeted modification. (III) Biodegradability and biocompatibility, especially for natural polymeric nanoparticles [56] (Fig. 5). Some synthetic polymers, such as polylactic acid (PLA), are also biologically safe and have been approved by the FDA for use in drug delivery and tissue engineering [57].

Some nanoparticles are able to polarise macrophages themselves without the need for reprogramming agents. Ann et al. found that both carboxyl-modified polystyrene nanoparticles and amino-modified nanoparticles successfully inhibited TAMs polarisation to M2 by downregulating the expression of CD200R, CD163 and IL-10, while effectively promoting M1 polarisation [58]. Parayath prepared HA-PEI nanoparticles loaded with miR125b and successfully promoted TAM polarisation to the M1 type after intraperitoneal injection into tumor-bearing mice [59]. Wang et al. prepared a shrinking polymer carrying the CSF1R inhibitor BLZ-945, which successfully reduced CD206 from 40 to 15%, while IL-12 and IFN-γ in TME increased to three times that of the control, effectively promoting M1-type TAM polarisation [60].

In recent years, membrane coating technology has been widely used, and some cell membranes can become specific drugs that affect macrophage polarisation. For example, the cell membranes of natural killer cells (NKs) and THP1 macrophages were coated with poly(lactic-co-glycolic acid) (PLGA) nanoparticles, resulting in tenfold higher IL-6 than controls [61]. DaSilvaa used PLGA as a degradable matrix core to deliver TLR7/8 agonist (TLR7/8 agonist resiquimod, R848), TLR3 agonist poly(I:C) and macrophage inflammatory protein-3 alpha -MIP3α, the combination of which significantly enhanced the anti-tumour effects of cancer vaccines [62]. Nam et al. used cationic polyethyleneimine (PEI) to absorb CpG and neoantigenic peptides to form a composite nanovaccine that could increase the amount of CD86 + M1 in the TME while decreasing CD206 + M2 [63].

Polymeric nanocarriers, such as N-(2-hydroxypropyl) methacrylamide (HPMA) copolymers, can avoid the systemic toxicity associated with conventional chemotherapy by targeting drug delivery to solid tumours. Melissa applied HPMA copolymers in an in vivo mouse model of metastatic breast cancer and observed HPMA copolymer uptake by CD68 + TAMs in primary and metastatic breast tumour specimens, demonstrating the potential use of this copolymer to preferentially target TAMs in the TME [64]. Zhang et al. injected M1 polarisation-related mRNA and diglycan-modified polymeric nanoparticles into three tumour models (ovarian cancer, melanoma and glioblastoma) and found that the nanoparticles could target macrophage CD206 receptors, thereby increasing the proportion of M1 TAM, inducing anti-tumour immunity and promoting tumour degeneration [65]. Wei developed two targeted polymer microspheres to deliver R837 and the anticancer drug doxorubicin to TAMs and tumour cells by intratumoral and intravenous injections, respectively, and these microspheres released R837 in tumour tissues to bind to TLR-7 receptors on TAM endolysosomal membranes and stimulate TAM maturation, thereby inducing anti-tumour immune response and alleviating immunosuppression in TME [66].

#### Inorganic nanoparticles

Inorganic nanomaterials are defined as nanoparticles composed of inanimate materials and typically include metallic nanomaterials, inorganic oxide nanomaterials and inorganic semiconductor nanomaterials [67]. Inorganic nanoparticles have many advantages, including: (I) Easy long-term preservation and tolerance to strict sterilisation conditions. (II) Finely controllable structure and shape. (III) Application in various microenvironments due to the unique physical, electrical, magnetic and optical properties of the substrate [68, 69].

**Fig. 6:**
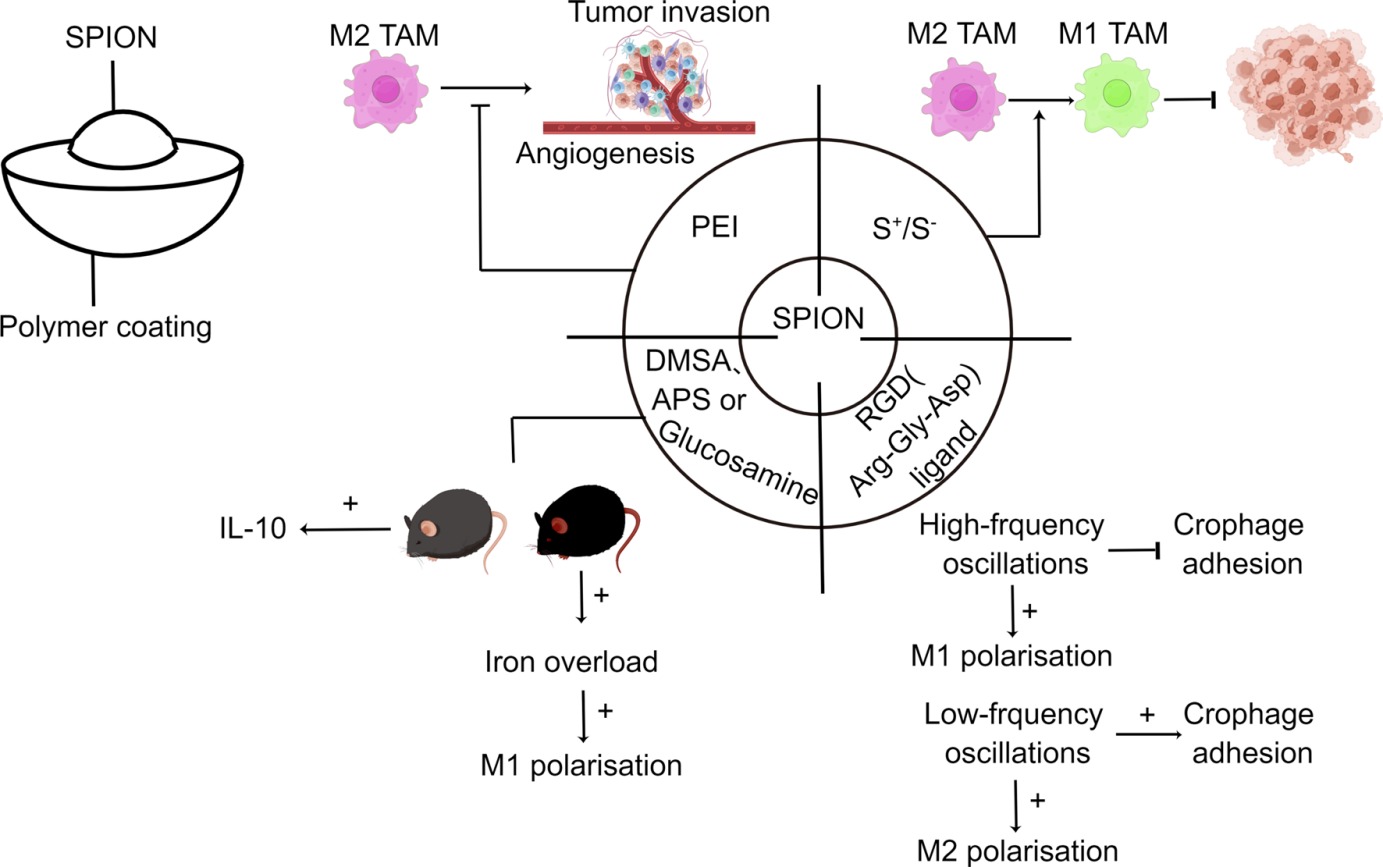
Functions and characteristics of inorganic nanoparticles. (By Figdraw) SPIONs: superparamagnetic iron oxide nanoparticles; PEI: polyethyleneimine; DMSA: dimercaptosuccinic acid; APS: astragalus polysaccharide; RGD: composed of arginine, glycine and aspartate

As mentioned above, some nanoparticles have the ability to reprogramme macrophages, and iron oxide nanomaterials are one of them [70]. The FDA has approved the iron supplement fermoxytol for the treatment of iron deficiency. Zanganeh, S. found through in vitro and mouse model studies that fermoxytol promotes TAM polarisation towards M1, thereby inhibiting tumour growth [71]. Mikhaylov et al. loaded the small molecule broad-spectrum inhibitor JPM-565 together with iron oxide nanoparticles into PEGylated liposomes to create "iron liposomes", resulting in local tissue protease inhibition and ultimately reduced breast cancer growth [72]. Vladimir found that polyethyleneimine (PEI)-coated superparamagnetic iron oxide nanoparticles (SPIONs) are biologically active nanosystems that limit tumour cell invasion by inhibiting the pro-angiogenic effect of M2-type TAM [73] (Fig. 6). Zhang's group discovered the importance of the surface charge of SPIONs in the phenotypic polarisation of macrophages and that both S + and S− significantly repolarised TAMs and inhibited tumour growth compared to neutral SPIONs (Fig. 6). Rojas et al. prepared SPIONs coated with dimercaptosuccinic acid, aminopropylsilane or aminodextran; in two M2 TAM models (mouse primary IL-4-activated bone marrow-derived macrophages and human M2-like differentiated THP-1 cells), SPION treatment altered their M2 activation profile and promoted IL-10 production [74] (Fig. 6). Kang et al. fabricated SPIONs containing RGD (composed of arginine, glycine and aspartate) ligands and remotely manipulated the nano-oscillations of magnetically responsive adhesion ligands by adjusting the oscillation frequency of the magnetic field. High frequency oscillations inhibit macrophage adhesion and promote the M1 polarisation phenotype, while low frequency oscillations do the opposite [75] (Fig. 6). Kang et al. fabricated SPIONs containing RGD (composed of arginine, glycine and aspartate) ligands and remotely manipulated the nano-oscillations of the magnetic field.

Li et al. prepared albumin-modified gold nanorods (AUNRs) using PTX and found that they could inhibit M2 polarisation and regulate the TME in tumour-bearing mice [76]. Manganese dioxide nanoparticles can be used to deliver repolarising agents. Liu. et al. prepared a nanodrug with a core of manganese dioxide and modified it with hyaluronic acid (HA): Man-HA-MnO2NPs. In vitro, M2 TAMs generated from RAW264.7 cells were co-incubated with Man-HA-MnO2NPs and it was found that M2 TAMs could be successfully converted to the M1 phenotype, which was also validated in a mouse model [77]. Furthermore, since calcium ions themselves can promote the production of pro-inflammatory cytokines such as IL-1β [78], calcium and some catalytic nanoparticles have also been shown to modulate the phenotype of macrophages. He et al. used peptide-modified calcium carbonate to deliver HA and IL-12-encoded plasmid DNA, and several M1 markers were increased in J774A.1 cells [79]. Zhang, X. found that black phosphorus nanoparticles [80] and mesoporous Prussian blue [81] could also be loaded with low molecular weight HA to polarise M2 to M1. Xu et al. stimulated the release of cytokines from tumour cells and TAMs using a combination of nanodiamond doxorubicin (Nano-dox) and the PD-L1 blocker BMS-1 to promote the polarisation of TAMs towards the M1 type [82]. Chen et al. developed a fibrin gel encapsulated with calcium carbonate nanoparticles loaded with anti-CD47 antibody to polarise TAMs towards M1-like phenotype, thereby enhancing the anti-tumour effect of macrophages [83]. Although many results demonstrate the feasibility of targeting TAM polarisation with inorganic nanomaterials, further studies are needed to verify their toxic effects and efficacy in real clinical settings.

#### Other nanomaterials

Through extensive studies of nanoparticles, researchers have developed many novel nanomaterials that target TAM polarisation. Cao et al. investigated an (EV)-like ginseng-derived nanoparticle (GDNP) and found that it could reverse M2-like polarisation in vivo and in vitro, thereby inhibiting tumour cell proliferation [84]. Cholesterol-modified Prussian blue nanoparticles were reported to inhibit tumour cells by targeting macrophages in lymph nodes through subcutaneous injection and polarising TAMs towards M1 [85]. Guo et al. designed GSH and reactive oxygen species (ROS) dual-responsive targeting TAMs and synthesised oligohyaluronic acid-mannose-folic acid (oHA-Man-FA, HMF) and astragalus polysaccharide-dithiodipropionic acid-paeoniflorin (APS-S-S-Pae, ASP), two hybrid materials that can self-assemble in water to form hybrid nanoparticles (HP-NPs@PTX/Bai). Such hybrid nanoparticles can be specifically released in the tumour microenvironment, remodel TAMs to the M1 phenotype and deliver the anti-tumour drugs PTX and Bai to the tumour site [86]. Deng. et al. extracted cuttlefish ink nanoparticles (CINPs) with significant antitumour efficacy. By activating the mitogen-activated protein kinase (MAPK) signalling pathway, CINPs can effectively reprogramme TAMs from M2 to M1 type [87].

### Photodynamic-assisted macrophage polarization

Photodynamic therapy (PDT) is a treatment that combines a photosensitiser (PS) with light irradiation at specific wavelengths to generate ROS to destroy cancer cells, and has been widely studied as a non-invasive treatment modality in cancer therapy. On the one hand, photodynamic effects can lead to an increase in ROS, disrupting intracellular homeostasis and remodelling TAM into M1-type [88, 89]. The main cascade activated by ROS signalling is the classical DNA damage response, in particular the ATM-CHK2 axis. As shown by Western blot, the ROS → ATM → CHK2 axis is indeed involved in the M1 polarisation of macrophages. That is, after ROS production, it leads to CHK2 activation, and the activated CHK2 affects metabolic reprogramming through activation of PKM2, which in turn affects the cell cycle through p21, and finally leads to M1 polarisation of macrophages [90]. On the other hand, dead tumour cells can induce the activation of APC to stimulate the production and proliferation of tumour-specific effector T cells [91, 92].

Yu, T.-T. et al. found that chlorin e6 E6 (Ce6) PDT can activate the stimulator of interferon genes (STING) molecule, which activates NF-κB-mediated nuclear polarisation and converts macrophages to the M1 phenotype [93]. They also found that lung cancer cell lines respond to Ce6, which disrupts the tumour microenvironment and induces conversion of macrophages to the M1 phenotype, thereby inhibiting lung cancer cell proliferation [94]. Kataoka, H. developed the photosensitiser mannose-conjugated (tetrafluorophenyl) chlorin (M-chlorin) and confirmed that M-chlorin PDT induced only M2 TAM death, altered the M1/M2 ratio and inhibited cancer cell proliferation [95]. Zhao et al. found that coating polyethylene glycol-modified graphene oxide GO-PEG with HPPH can increase the solubility of the photosensitiser and enhance the uptake of HPPH by TAM, thus improving the photodynamic efficacy [96]. Similarly, Yawen Wei. et al. prepared a particle (m@Au-D/B) loaded with the drug doxorubicin and l-buthionine sulfoximine (BSO) with cancer cell membrane artefacts, which could stimulate photothermal effects and catalyse to produce more ROS under photodynamic forces and promote TAM polarisation towards M1 phenotype [97]. Rong et al. found that melanin-like PDA-induced PTT could partially eliminate solid tumours by hyperthermia, while tumor-associated antigens (TAAs) released by hyperthermic cell death could inhibit cancer cell proliferation by exploiting the antigen-presenting ability of cells to promote TAM polarisation towards M1 and continuously increase cytotoxic T lymphocyte infiltration at tumour sites [98].

### Other methods to polarize macrophages

Epidemiological evidence has shown that statins are associated with beneficial outcomes in patients with brain tumours [99], and statins have been reported to reconstitute the tumour microenvironment and repolarise TAM function [100, 101]. Simvastatin (SV) is a commonly used cholesterol-lowering drug that converts TAM from M2 to M1 type. Accordingly, Yin et al. prepared a T12/PD-L1-Nb double-modified liposome for the treatment of small cell lung cancer and achieved good efficacy [102]. Jin et al. found that SV reversed EMT in cancer cells and inhibited tumour progression by inhibiting cholesterol homeostasis and repolarising M2 to M1 type [103]. Tu et al. developed a deformable liposome system (D-Lipo) for co-delivery of vorinostat and simvastatin to remodel TME with anti-tumour mechanisms including M2 TAM repolarisation, anti-angiogenesis and TME remodelling. The results showed that the increased number of anti-tumour M1 macrophages and CD8 + T cells provided a promising pathway for epigenetic drugs [104].

Iron plays an important role in macrophage polarisation by altering metabolism and redox status. However, the effect of iron on the immune status of macrophages remains controversial. Choi, E. J. found that ferric ammonium citrate (FAC) could alter the phenotype of macrophages and induce them to polarise towards M1 [105]. Yang found that iron hydride released large amounts of Fe2 + under normal blue light irradiation, and by intravenous pre-injection of tumor-bearing mice, sodium iron hydride induced tumor-associated macrophage (TAM) polarisation from the tumor-promoting M2 type to the tumor-killing M1 type. The combined effect of the light/Fe2 + pathway attenuated lung metastasis in mice [106]. In recent years, magnetothermal therapy (MTT) has become a clinical treatment for malignant tumours. Magnetothermal therapy promotes the shift of TAM from a polarised M2 phenotype to a pro-inflammatory M1 phenotype, while enhancing T lymphocyte infiltration into the TME, thereby inhibiting tumour proliferation [107].

## Summary and perspective

There are not many reports on the use of the above research in the clinic, but we need to consider whether there are potential side effects or whether certain drug resistances will develop. For example, adverse reactions to large deposits of nanomaterials in the body, and whether the ROS generated by PDT cause any damage to the body. All of these issues deserve further attention and consideration. Currently, the FDA has approved more than 30 NP agents for therapeutic or diagnostic use, including metal NPs, albumin NPs and liposomes, many of which have applications in cancer therapy [108]. Polylactic acid (PLA), polyglycolic acid (PGA) and polylactic acid (PLGA) have been approved by the US Food and Drug Administration (FDA) [109]. Among these, PLGA is one of the most successfully developed polymers and has been widely used for extensive cancer therapy due to its targeting and TAM polarisation towards M1. With the discovery of the important role of macrophages in the innate and adaptive immune system, the advent of nanomedicines and other related therapies that specifically target TAM polarisation has opened new avenues for immunotherapy of tumours. This is further supported by recent studies showing that M1-like genetically modified macrophages are able to activate immature dendritic cells and induce T lymphocyte infiltration into the TME, thereby inhibiting tumour proliferation [110]. With all these findings in TAM, we anticipate further clinical translational contributions of TAM polarisation-related therapies in this emerging field. However, the emergence of new therapeutic approaches often brings more opportunities and challenges. Considering that existing TAM have emerged as a subset of TAM beyond the M1/M2 classification, whether TAM can be further classified, how the size of the weight of each type of TAM varies in different types of tumours, whether the proportion of each type of TAM in the tumour is different, and what are the side effects of the different methods of polarising TAM mentioned in the article, are all worthy of further consideration.

## Data Availability

No datasets were generated or analysed during the current study.
